# Effects of genetic variations on microRNA: target interactions

**DOI:** 10.1093/nar/gku675

**Published:** 2014-07-31

**Authors:** Chaochun Liu, William A. Rennie, C. Steven Carmack, Shaveta Kanoria, Jijun Cheng, Jun Lu, Ye Ding

**Affiliations:** 1 Wadsworth Center, New York State Department of Health, Center for Medical Science, 150 New Scotland Avenue, Albany, NY 12208, USA; 2 Department of Genetics and Yale Stem Cell Center, Yale University, New Haven, CT 06520, USA

## Abstract

Genetic variations within microRNA (miRNA) binding sites can affect miRNA-mediated gene regulation, which may lead to phenotypes and diseases. We perform a transcriptome-scale analysis of genetic variants and miRNA:target interactions identified by CLASH. This analysis reveals that rare variants tend to reside in CDSs, whereas common variants tend to reside in the 3′ UTRs. miRNA binding sites are more likely to reside within those targets in the transcriptome with lower variant densities, especially target regions in which nucleotides have low mutation frequencies. Furthermore, an overwhelming majority of genetic variants within or near miRNA binding sites can alter not only the potential of miRNA:target hybridization but also the structural accessibility of the binding sites and flanking regions. These suggest an interpretation for certain associations between genetic variants and diseases, i.e. modulation of miRNA-mediated gene regulation by common or rare variants within or near miRNA binding sites, likely through target structure alterations. Our data will be valuable for discovering new associations among miRNAs, genetic variations and human diseases.

## INTRODUCTION

Genetic variations within gene regulatory elements may affect gene expression levels in an allele-specific manner and thereby contribute to the variation in complex human phenotypes and diseases. Many disease-associated regulatory polymorphisms such as variants in *cis*-elements (1,2) operate at the stage of transcriptional regulation through altering the binding affinity of transcription factors. Recently, polymorphisms within post-transcriptional regulatory elements in particular microRNA (miRNA) binding sites have been studied (3,4). These polymorphisms also represent an important class of genetic variations.

miRNAs are an abundant class of small endogenous non-coding RNAs of ∼22 nucleotides (nts) in length. More than 1,000 human miRNAs have been discovered (5), while more than 30% of human protein-coding genes are predicted to be regulated by miRNAs (6). miRNAs are key post-transcriptional regulators involved in diverse developmental processes, molecular and cellular pathways and human diseases (7). A mature miRNA can guide RNA-induced silencing complex for target recognition through hybridization between the miRNA and the cognitive messenger RNAs (mRNAs). Successful target binding usually results in translational repression and/or mRNA degradation (8). It has been demonstrated that genetic variants within miRNA binding sites can modulate gene expression and protein output levels and affect phenotypes or cause disease (3,4,9–15). Several studies have systematically identified the genetic variants within human miRNA target sites (16–21) and performed part or all of the following analyses: (i) investigation of natural selection via statistical analysis of the frequency of single nucleotide polymorphisms (SNPs) within miRNA seed (2–7 nt) complementary regions, miRNA binding sites and the 3′ untranslated regions (3′ UTRs) of mRNAs or the entire mRNAs (17–21); (ii) measurement of the SNP-induced effect on miRNA binding by hybridization energy change (16,17); and (iii) association of the miRNA-related SNPs with human phenotypes or diseases (16,17,20). For some of these studies, a small fraction of miRNA binding sites were experimentally validated. The remaining miRNA binding sites in all of these studies were identified by computational predictions that can have high numbers of false positives or false negatives (22). Inaccuracy in predictions may bias such analyses. Moreover, these studies only considered common genetic variants with minor allele frequencies (MAFs) greater than or equal to 1% or 5%. Although common genetic variants have been a focus of disease association studies, some rare variants may have significant impact on an individual's risk of certain phenotypes or diseases (23–28). To date, there has been a lack of systematic studies that include both common and rare genetic variants, as well as variants without frequency information. Genetic variations may alter the local secondary structure of mRNA sequences (29). A change in structural accessibility can affect target recognition by miRNAs (30–33). However, the hybridization energy used in the previous studies (16,17) does not measure the effect of local target structure change induced by genetic variants within the binding sites. In this work, we consider several target structure features for measuring the effects of variants on local target structure. Moreover, two SNPs near miRNA binding sites were reported to lead to either a change in local target secondary structure (34) or an alteration of miRNA regulation (35). We here systematically study such effects of SNPs in the flanking regions of miRNA binding sites.

A human miRNA interactome of ∼18,500 miRNA:mRNA interactions has been experimentally identified by CLASH (36). The CLASH technique performs high-throughput crosslinking, ligation and sequencing of miRNA-target RNA duplexes associated with human AGO1. It allows direct observation of miRNA:target interactions revealed by CLASH chimeras. Over 98% of the interactions were formed *in vivo* in human cells (36). The CLASH study has presented a high-quality data set of high-confidence miRNA binding sites, which enables accurate identification of genetic variants within or near true miRNA binding sites. Thus, this data set provides a solid foundation for a systematic investigation of the effects of miRNA-related variations on miRNA-mediated gene regulation and human phenotypes or diseases. To pursue this objective, we start with a comprehensive transcriptome-wide survey on natural selection for genetic variants within miRNA binding sites identified by CLASH. In addition to hybridization energy, we consider four features to measure the effects of genetic variants within or near miRNA binding site on local target structure and the potential of miRNA:target hybridization. Furthermore, we identify miRNA-related genetic variants for cancer genes and also those associated with known human phenotypes or diseases to facilitate further studies on individual susceptibility to complex diseases.

## MATERIALS AND METHODS

### Data processing

We downloaded the human genetic variant data set ‘phase1_integrated_release_version3’ from 1000 genomes FTP (ftp://ftp.1000genomes.ebi.ac.uk/vol1/ftp/release/20110521/) which contains phased genotype calls on 1,092 human samples for SNPs, short indels and large deletions. These variants were mapped to Ensembl transcriptome of all protein-coding genes using the annotation file (Ensembl Release 60) from Ensembl genome browser (http://www.ensembl.org).

The CLASH data set includes ∼18,500 miRNA:target interactions as chimeric sequencing reads for 399 miRNAs and 7,390 transcripts. Each chimeric read contains one miRNA and a target-binding region of 42–119 nts in length. The miRNA binding sites within the CLASH chimeras are identified by the RNAhybrid program (37) for either seed sites (i.e. canonical sites) or seedless sites (i.e. noncanonical sites). Seed sites include 8mer, 7mer-A1, 7mer-m8, 6mer and offset 6mer sites (38). RNAhybrid also presents the conformation of the miRNA:target hybrid in addition to the start and end nucleotide positions of the binding site. For each binding site, we calculated conservation score as the average of individual nucleotide conservation scores from the UCSC genome browser. These scores were generated by the PhastCons program (39) through multiple-sequence alignments of nine primate genomes to the human genome (hg19).

### Variant frequency analysis

For the subset of common variants and the subset of rare variants, we first counted the numbers of variants residing within the entire mRNA, coding sequence (CDS), or 3′ UTR for the whole transcriptome, CLASH transcripts and miRNA binding sites, respectively. A transcript is referred to as a CLASH transcript if it is represented by at least one CLASH chimera. The 5′ UTR was not included in region-specific analysis, due to sparse variant data. We next computed the length for each of these regions. The variant density for a region was computed by the number of variants divided by the length of the region. The *P*-value from Fisher's exact test (40) was used to evaluate the significance of the difference in variant densities.

### Thermodynamic and target structure features for measure of variation effects

In addition to ΔG_hybrid_, a measure of hybrid stability computed by RNAhybrid (37), we consider four other thermodynamic and target structure features. ΔG_total_, a measure of total energy change, is the key characteristic of a two-step model for miRNA:target hybridization (33) and can be considered as a measure of potential for successful miRNA:target hybridization. We also computed three probabilistic measures of structural accessibility for the miRNA binding site, the 25-nt blocks upstream and downstream of the site as follows. For a block of nucleotides, the accessibility was computed by the average probability that each nucleotide in the block is single-stranded, based on the RNA secondary structure sampling algorithm implemented by Sfold (41,42).

For measuring the effects of genetic variants, ΔΔG_hybrid_ was computed by [ΔG_hybrid_ (mutant) − ΔG_hybrid_ (wild type: WT)] to measure the variant effect on hybrid stability. ΔΔG_total_ was computed by [ΔG_total_ (mutant) − ΔG_total_ (WT)] to measure the variant effect on the potential of miRNA:target hybridization. Δsite_access was computed by [site accessibility (mutant) − site accessibility (WT)] to measure the variant effect on structural accessibility of the miRNA binding site. Similarly, Δupstream_access and Δdownstream_access were computed to measure the variant effects on structural accessibility of the 25-nt blocks upstream and downstream of the binding site, respectively.

### Identification of miRNA-related variants for cancer genes or associated with diseases

We downloaded the list of cancer genes from the CancerGenes database (43). Using this list, we identified all miRNA-related variants in cancer genes that reside either in the miRNA binding sites or in the 25-nt flanking regions. Although the genome-wide association studies (GWAS) have identified many genetic variants associated with diseases, very few miRNA-related variants in this study can be found or corroborated by GWAS results. We thus performed a literature search using PMC databases to retrieve articles reporting the association between each of these miRNA-related variants and human phenotypes or diseases, and collected the miRNA-related variants that were reported to be associated with human phenotypes or diseases in one or more studies.

## RESULTS

### Genetic variation frequency in different regions

We identified 955,275 variants across 75,853 Ensembl transcripts. These include 302,797 (31.7%) variants with MAF ≥1% and 652,478 (68.3%) variants with MAF <1%. Among all of the miRNA binding sites from CLASH chimeras, ∼81.3% are seedless according to the definition of seed sites (38). Comparisons were made between whole transcriptome (defined by Ensembl transcripts) and CLASH transcripts, and between CLASH transcripts and miRNA binding sites within CLASH chimeras.

**Figure 1.**
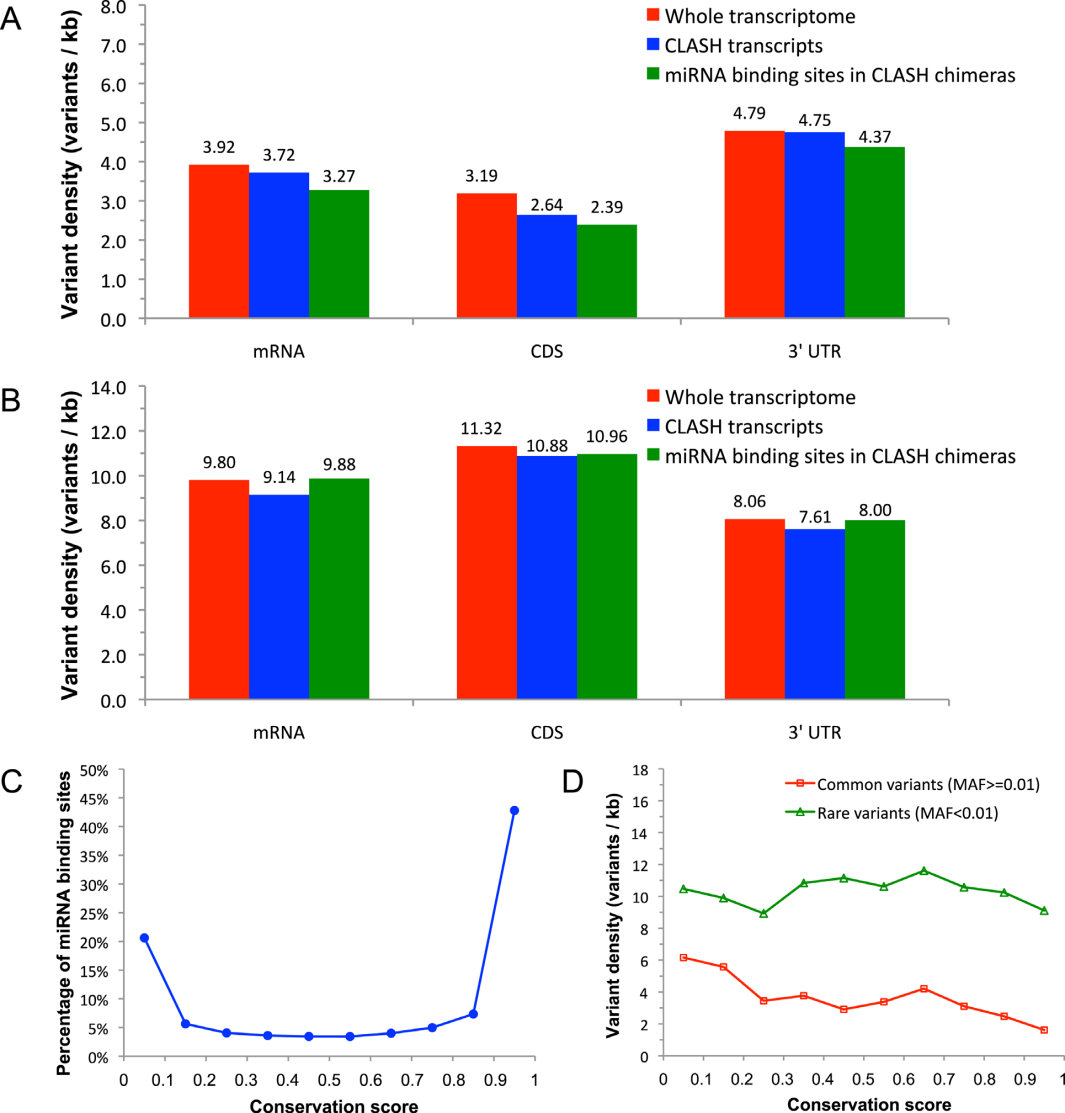
Variant densities in whole transcriptome, CLASH transcripts and miRNA binding sites for (**A**) common variants (MAF ≥ 1%); (**B**) rare variants (MAF <1%); (**C**) percentages of miRNA binding sites by evolutionary conservation levels; (**D**) density of variants (common or rare) with different MAF thresholds for miRNA binding sites grouped by conservation level.

We focus on presenting results using the common MAF thresholds of 1% defining common variants and rare variants. The conclusions are generalizable to a wide range of thresholds (Supplementary Figure S1). For both common and rare variants in the three target regions (mRNA, CDS and 3′ UTR), the densities for CLASH transcripts (blue bars in Figure 1A and B) are significantly lower than those for the whole transcriptome (red bars in Figure 1A and B), with all *P*-values under 0.04 for density comparisons. It indicates that miRNA binding sites are more likely to reside within those targets in the transcriptome with lower variant densities, consistent with a previous study (21). In all of the three target regions (mRNA, CDS and 3′ UTR), for common variants, the densities for miRNA binding site (green bars in Figure 1A) are significantly lower than those for the CLASH transcripts (blue bars in Figure 1A), with all *P*-values under 0.03. For rare variants, the densities for miRNA binding site (green bars in Figure 1B) are marginally higher than those for the CLASH transcripts (blue bars in Figure 1B). These suggest miRNAs binding sites are more likely to reside within target regions in which nucleotides have low mutation frequencies. Moreover, for common variants, the densities for 3′ UTRs are substantially higher than those of CDSs (Figure 1A); for rare variants, the densities for CDSs are substantially higher than those of the 3′ UTRs (Figure 1B). These indicate that rare variants tend to reside in CDSs, whereas common variants tend to reside in the 3′ UTRs. This may be due to the fact that under codon constraints, CDS tends to be more conserved than 3′ UTR.

We estimate that ∼43% of miRNA binding sites in the CLASH chimeras are highly conserved (conservation score >0.9), while ∼21% are poorly conserved (conservation score ≤0.1) (Figure 1C). This suggests that while many miRNA binding sites are conserved, a significant portion are species specific. For highly conserved sites, the variant density is significantly lower than that of other sites (*P*-value = 8.1e−28). Especially for common variants, the densities generally decrease with increasing conservation (Figure 1D). These findings are consistent with previous observations on predicted conserved miRNA binding sites (18).

### Effects of genetic variants within miRNA binding sites on miRNA:target interaction

Genetic variants within miRNA binding sites can have impact on miRNA:target hybridization through either altering local target structure and accessibility or disruption/creation of base pair(s). ΔΔG_hybrid_ (see the MATERIALS AND METHODS section) was used in a previous study (17) to measure the effects of genetic variants on stability of the miRNA:target hybrid. A positive value of ΔΔG_hybrid_ indicates a decrease in hybrid stability due to the variant, whereas a negative value indicates an increase in hybrid stability. However, ΔG_hybrid_ does not measure target structural accessibility and the potential of miRNA:target hybridization. Here we compute four features ΔΔG_total_, Δsite_access, Δupstream_access and Δdownstream_access (see the MATERIALS AND METHODS section) for measuring the variant effects on target structure accessibility and the potential of miRNA:target hybridization. A positive ΔΔG_total_ indicates a decrease in the potential of miRNA:target hybridization due to the variant, whereas a negative value indicates an increase in the potential. A positive value for Δsite_access, or Δupstream_access or Δdownstream_access indicates increased structural accessibility at the miRNA binding sites or the flanking region(s), whereas a negative value indicates decreased accessibility. A larger change in any of the energetic or accessibility measures above could have a greater impact on miRNA:target interactions. The values of ΔG_hybrid_, ΔG_total_, site_access, upstream_access and downstream_access for both wild type miRNA:target interactions and mutant miRNA:target interactions can be found in Supplementary Table S1, together with seed type, maximal length of continuous Watson–Crick base-pairing in the region from miRNA nucleotide 12 to the 3′ end, predicted conformation of miRNA:target hybrid for each miRNA binding site of CLASH chimeras and the GO categories for cancer genes. We identified a total of 4109 variants residing within miRNA binding sites. For MAF threshold of 1%, there are 1047 common variants and 3062 rare variants. In general, the histograms representing distributions of effect measures for common variants are similar to those for rare variants.

**Figure 2.**
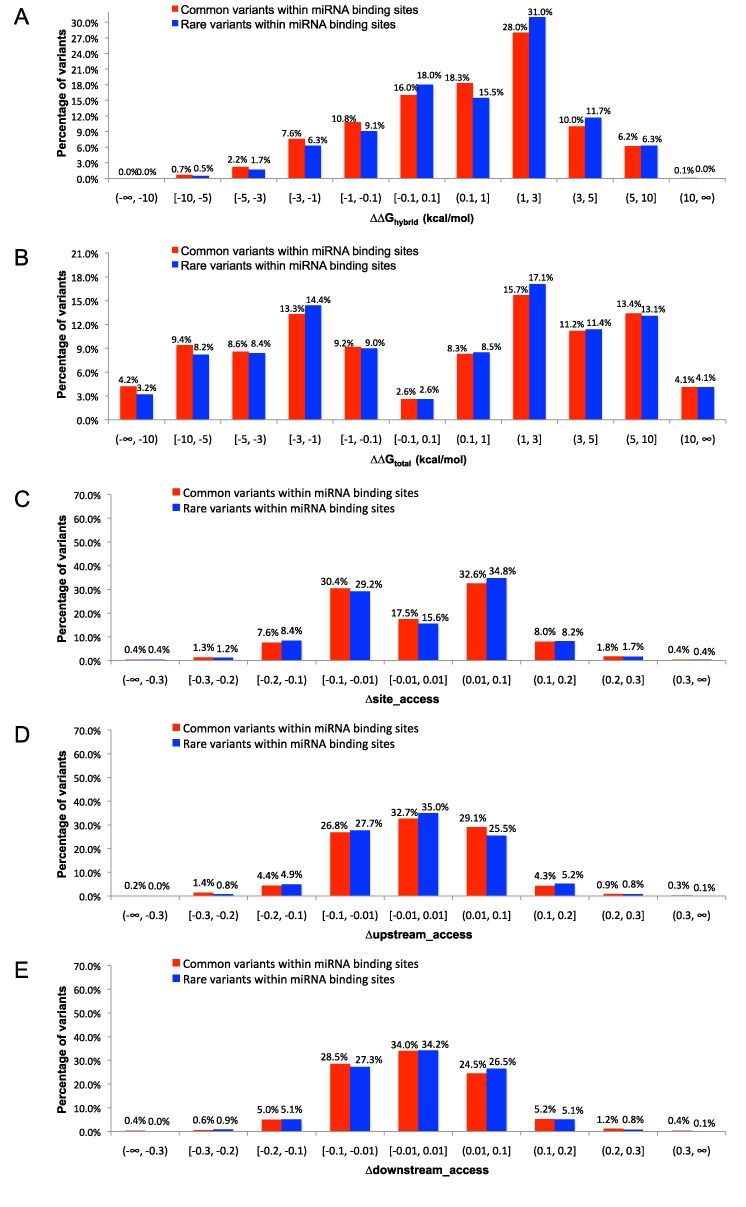
The histograms of effect measures for common (MAF ≥1%) and rare (MAF <1%) variants in miRNA binding sites (the horizontal axis intervals (a,b], [a,b), (a,b), [a,b] are defined by a<*x*≤b, a≤*x*<b, a<*x*<b, a≤*x*≤b, respectively, where *x* is the value of the feature). (**A**) ΔΔG_hybrid_; (**B**) ΔΔG_total_; (**C**) Δsite_access; (**D**) Δupstream_access; (**E**) Δdownstream_access.

The histograms of ΔΔG_hybrid_ for common and rare variants in miRNA binding sites are shown in Figure 2A. For common variants, 44.3% decrease and 10.5% increase the hybrid stability by at least 1 kcal/mol. For rare variants, 49% decrease and 8.5% increase the hybrid stability by at least 1 kcal/mol. By varying the change in hybrid stability, we also present cumulative distributions of absolute value of ΔΔG_hybrid_ for common and rare variants in miRNA binding sites (Supplementary Figure S2A). These indicate that a majority of variants in miRNA binding sites can alter hybrid stability. In particular, both common and rare variants tend to weaken the miRNA:target hybrid stability (*P*-values <2.3e−45).

The histograms of ΔΔG_total_ for common and rare variants in miRNA bindings sites are shown in Figure 2B. For common variants, 44.4% decrease and 35.5% increase the hybridization potential by at least 1 kcal/mol, respectively. For rare variants, 45.7% decrease and 34.2% increase the hybridization potential by at least 1 kcal/mol, respectively. By varying the change in the total hybridization energy, we also present cumulative distributions of absolute value of ΔΔG_total_ for common and rare variants in miRNA binding sites (Supplementary Figure S2B). Noticeably, even with a relatively high threshold of |ΔΔG_total_|, such as 5 kcal/mol, a substantial fraction (∼30%) of variants changed total hybridization energy. These indicate that a majority of variants in miRNA binding sites can alter hybridization-potential. In particular, both common and rare variants tend to reduce the potential of miRNA:target hybridization (*P*-values <2.8e−4).

The histograms of Δsite_access, Δupstream_access and Δdownstream_access for common and rare variants in miRNA binding sites are shown in Figure 2C–E. For common variants, ∼82% can alter structural accessibility of the miRNA binding sites (with a cutoff of 0.01); 67% can alter the accessibility of the upstream region of 25 nts; and 66% can alter downstream accessibility. Comparable percentages were observed for the rare variants. By varying the change in accessibility, we also present cumulative distributions of absolute values of Δsite_access, Δupstream_access and Δdownstream_access, for common and rare variants in miRNA binding sites (Supplementary Figure S2C–E). These indicate that for variants in miRNA binding sites, the majority can alter structural accessibility of the miRNA binding sites and the flanking regions, thereby affecting the access to the target by miRNA–Argonaute complex. Moreover, we computed the mean MAF for variants (in miRNA binding sites) which affect site accessibility (|Δsite_access|>Θ) and those which do not (|Δsite_access|≤Θ), where Θ is a threshold varying from 0.01 to 0.2. We observed that variants which affect site accessibility have substantially lower mean MAF than those variants which do not (Supplementary Figure S4A). This indicates that variants (in miRNA binding sites) which affect site accessibility tend to have lower frequencies for minor alleles.

### Effects of genetic variants near miRNA binding sites on miRNA:target interaction

Genetic variants within flanking regions of miRNA binding sites may also alter local target structure, affecting the potential of miRNA:target hybridization. Because these variants reside outside miRNA binding sites, ΔG_hybrid_ is the same for all alleles (i.e. ΔΔG_hybrid_ = 0) and thus is not useful for analysis of their effects. Therefore, we only consider the four structural features. Some variants (e.g. insertions or deletions of multiple nucleotides) can substantially change a flanking region. To facilitate analysis on flanking regions of a pre-specified length, we focus on SNP variants within a 25-nt block either upstream or downstream of the miRNA binding sites. For MAF threshold of 1%, we identified 1234 common SNPs and 3778 rare SNPs for upstream regions (Supplementary Table S2), and 1231 common SNPs and 3746 rare SNPs for downstream regions (Supplementary Table S3). Generally, the histograms of effect measures for common variants are very similar to those for rare variants. The values of the structural features for both the wild-type miRNA:target interactions and mutant miRNA:target interactions are also given in Supplementary Tables S2 and S3.

**Figure 3.**
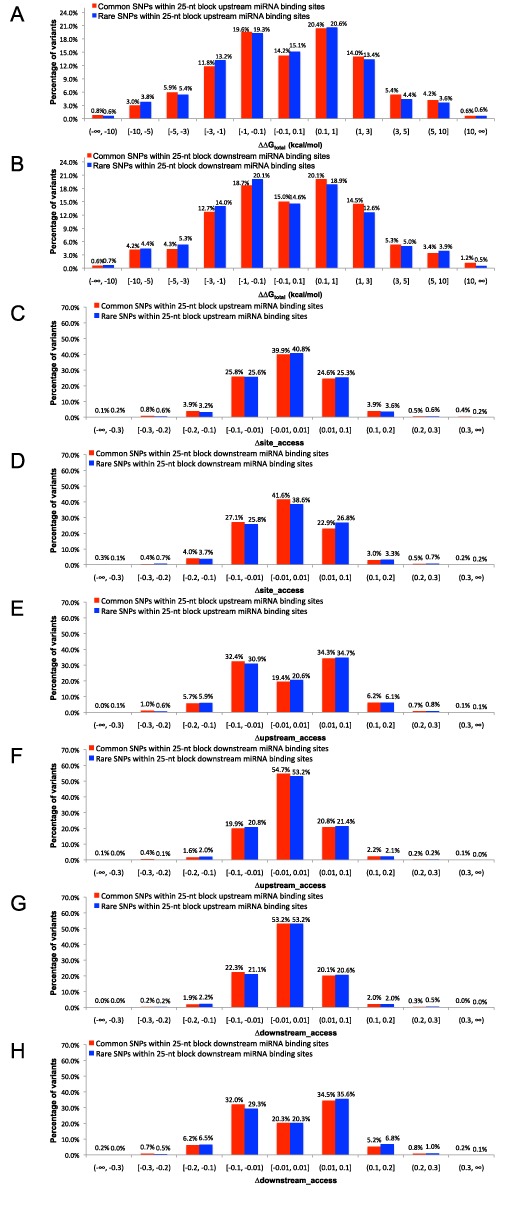
The histograms of effect measures for common (MAF ≥1%) and rare (MAF <1%) SNPs in 25-nt blocks upstream or downstream of miRNA binding sites. (**A**) ΔΔG_total_ for SNPs upstream of sites; (**B**) ΔΔG_total_ for SNPs downstream of sites; (**C**) Δsite_access for SNPs upstream of sites; (**D**) Δsite_access for SNPs downstream of sites; (**E**) Δupstream_access for SNPs upstream of sites; (**F**) Δupstream_access for SNPs downstream of sites; (**G**) Δdownstream_access for SNPs upstream of sites; (**H**) Δdownstream_access for SNPs downstream of sites.

The histograms of ΔΔG_total_ for common and rare SNPs in upstream regions are shown in Figure 3A. Among common SNPs, 24.2% decrease and 21.5% increase the hybridization potential by at least 1 kcal/mol. A similar histogram is also shown for the rare SNPs. The results are similar for the downstream regions (Figure 3B). By varying the change in the total hybridization energy, we also present cumulative distributions of absolute value of ΔΔG_total_ for common and rare variants in the flanking regions of miRNA binding sites (Supplementary Figure S3A and B). Overall, about half of the SNPs in the flanking regions of miRNA binding sites can alter the potential of the miRNA:target hybridization by at least 1 kcal/mol, and by over 10 kcal/mol in some cases. The nearly symmetric distributions indicate that these SNPs are nearly equally likely to decrease or increase miRNA:target hybridization potential. Furthermore, the histograms have heavier weights in the center than those in Figure 2B, indicating that the effects of variants outside miRNA binding sites are more moderate than those within the binding sites.

The histograms of Δsite_access, Δupstream_access and Δdownstream_access for common and rare SNPs in either upstream or downstream regions of miRNA binding sites are shown in Figure 3C–H. The histogram distribution for Δsite_access shows that ∼60% of common or rare SNPs near miRNA binding sites can alter the structural accessibility of the binding sites (with a cutoff of 0.01), even though they reside outside the sites. For either common or rare SNPs in the upstream regions, ∼80% can alter the structural accessibility of the upstream regions; 47% can alter the downstream accessibility. For common or rare SNPs in the downstream regions, ∼46% can alter the structural accessibility of the upstream regions; 80% can alter the downstream accessibility. These percentages of the accessibility-altering variants suggest that the effects decrease with increasing distance from a region to the variant (i.e. 80% > 60% > 47% for upstream SNPs; 46% < 60% < 80% for downstream SNPs). By varying the change in accessibility, we also present cumulative distributions of absolute values of Δsite_access, Δupstream_access and Δdownstream_access for common and rare variants in the flanking regions of miRNA binding sites (Supplementary Figure S3C–H). Overall, these results indicate that majority of genetic variants near miRNA binding sites can alter the structural accessibility of both the binding sites and the flanking regions. Moreover, we computed the mean MAF for variants (in 25-nt flanking regions of miRNA binding sites) which affect site accessibility and those which do not, and observed the same result as for variants in miRNA binding sites (Supplementary Figure S4B). This indicates that among variants in 25-nt flanking regions of miRNA binding sites, those that affect site accessibility tend to have lower frequencies for minor alleles.

### Association of variants within miRNA binding sites with human diseases or phenotypes

Among the 4109 variants within miRNA binding sites (Supplementary Table S1), we identified 28 common variants and one rare variant that are associated with human diseases or phenotypes (Supplementary Table S4). A majority of these variants can substantially alter the potential of the miRNA:target hybridization. For example, the variant rs1049255 (G>A) in the 3′ UTR of gene *CYBA* was reported to be associated with NADPH oxidase (NOX) activity, oxidative stress and acute kidney injury (44). It leads to lower mRNA and protein expression of *CYBA* and reduced NOX activity (45). Interestingly, the allele A increases the potential of hybridization between *CYBA* and *miR-320a* by 4.3 kcal/mol. The lower levels of gene expression and NOX activity may be interpreted by enhanced regulation of *miR-320a* expressed in the kidney. We note that ΔΔG_hybrid_ is rather small in this case (−0.7 kcal/mol). Another example relates to the rare variant rs71653621 (A>G, MAF = 0.0014) in the CDS of gene *PARK7*, which was reported to cause early onset and familial Parkinson's disease (PD) (46). The report also showed that the mutation causes 1.3% decrease in *PARK7* mRNA folding energy compared to the wild-type sequence *in silico* and suggested a possible small effect on *PARK7* gene function (46). CLASH chimeras and our SNP analysis revealed an interaction of *miR-92b*:*PARK7* with rs71653621 residing within the miRNA binding site. The mutation increases the potential of hybridization between *PARK7* and *miR-92b* by 6.3 kcal/mol, suggesting possible effect on miRNA-mediated gene regulation. We note that the ΔΔG_hybrid_ value is also rather small in this case (0.3 kcal/mol). This presents a striking example of a rare genetic variant being associated with human disease.

### Association of SNPs near miRNA binding sites with human diseases or phenotypes

Among the 5012 SNPs in the 25-nt blocks upstream of miRNA binding sites (Supplementary Table S2), we identified 20 common SNPs and one rare SNP that are associated with human diseases or phenotypes (Supplementary Table S5). Among the 4977 SNPs in the 25-nt blocks downstream of miRNA binding sites (Supplementary Table S3), we identified 24 common SNPs and one rare SNP that are associated with human diseases or phenotypes (Supplementary Table S6). A majority of the upstream and downstream SNPs can substantially alter the potential of the miRNA:target hybridization. For example, the SNP rs2228075 (G>A) in CDS of gene *IMPDH1* is upstream of an *miR-615-3p* binding site identified by CLASH. This SNP was suggested to be adequate for the identification of patients at high risk of mycophenolate mofetil gastrointestinal intolerance (47). *miR-615-3p* was found to be expressed in colorectal cells (48). We observed large ΔΔG_total_ of about −5 kcal/mol for the allele mutation G>A, indicating a substantial enhancement of the potential of the hybridization between *IMPDH1* and *miR-615-3p* and potential decrease of *IMPDH1* expression level. It may provide an interpretation for the high risk of mycophenolate mofetil gastrointestinal intolerance, since *IMPDH1* is a regulation receptor in response to mycophenolate concentration (49). Another example relates to the SNP rs3088440 (C>T) in 3′ UTR of gene *CDKN2A*. This SNP is downstream of *miR-10b* binding site identified by CLASH and is associated with melanoma risk and second primary malignancy risk after index squamous cell carcinoma of the head and neck (50,51). For ΔΔG_total_, we observed a large value of 4.1 kcal/mol for *miR-10b*:*CDKN2A* hybridization.

## DISCUSSION

Previous studies were primarily based on predicted miRNA binding sites particularly seed sites, and in a few cases involved small numbers of validated miRNA binding sites. However, the reliance on the seed sites is a major limitation, because an overwhelming majority of predicted seed sites were not supported by the CLIP technique for miRNA binding identification (52). Furthermore, only ∼18.7% of the miRNA bindings sites from the CLASH chimeras are seed sites. To avoid potential biases, we based our analyses on the large set of miRNA binding sites experimentally identified by CLASH. In addition, previous work only examined common variants within miRNA binding sites. In this work, we also studied variants near miRNA binding sites as well as rare variants that may contribute to an individual's risk of certain phenotypes or diseases (23–28). Rare variants have been largely unexplored. Examination of the effects of miRNA-related rare variants complements existing techniques for the identification of candidate causal variants. The rare variants with large effects in this work could be promising candidates for causal variants in disease-association research.

It has been postulated that rare variants tend to have stronger biological effects while common variants tend to have weaker biological effects (53). This is consistent with our findings that rare variants tend to reside in CDSs, whereas common variants tend to reside in the 3′ UTRs. Rare variants could have greater biological effects by altering protein sequences, whereas common variants are often involved in post-translational regulation through regulatory regions in the 3′ UTRs. On the other hand, miRNA binding sites are more likely to reside within those targets in the transcriptome with lower variant densities, especially target regions in which nucleotides have low mutation frequencies.

Previous studies were limited to the miRNA:target hybrid stability measured by ΔG_hybrid_. This feature ignores the effects of local target structure that have been shown to be important for target binding by miRNAs (30–33). In addition, it is not useful for studying the effects of variants residing outside miRNA binding sites. To address these limitations, we considered four structure-based features. These features provide new insights into the effects of genetic variants on the potential of miRNA:target hybridization as well as the structural accessibility of both the binding site and flanking regions. Moreover, they also facilitate the examination of effects of genetic variants near miRNA binding sites. For the cases with disease associations examined here, we observed a substantial ΔΔG_total_, but rather small ΔΔG_hybrid_. This observation and the findings from the previous studies (29,34,54) suggest that alteration in local target structure can be an important mechanism for genetic variants to have biological effects, some of which are associated with diseases or phenotypes.

We identified a list of variants that are associated with human phenotypes and diseases, and showed that such associations could be interpreted by the effects of variants on target binding by miRNAs. The reliable large set of miRNA binding sites from CLASH and broad gene regulation by miRNAs make our comprehensive list of miRNA-related variants with their effect measures valuable for the discovery of new associations between genetic variations and human diseases or phenotypes. Our findings also present a general mechanistic interpretation for certain associations between genetic variants and diseases, i.e. modulation of miRNA-mediated gene regulation by common or rare genetic variants within or near miRNA binding sites. In particular, among our list of miRNA-related genetic variants within cancer genes, some may be promising candidates for causal cancer variants.

We have shown that the genetic variants within or near miRNA binding sites can affect miRNA:target interactions. Such miRNA-related variants can be reliably identified by using miRNA:target interactions directly observed by CLASH. Therefore, available associations between these variants and human diseases could be used to infer associations between miRNAs and human diseases. This provides a means for the identification of miRNAs as potential biomarkers for human diseases. Our data will facilitate such investigations.

CLASH provides much more accurate miRNA binding site information than CLIP methods (55,56), as the two RNA molecules are in close proximity to each other. Further, the strong binding energies of chimeric reads indicate that these have resulted from genuine RNA–RNA interactions rather than from proximity-induced ligation of non-interacting RNAs in solution. Additionally, control experiments indicated that almost >98% of the miRNA–target RNA interactions by CLASH had formed *in vivo* in human cells, ruling out the possibility of false interactions that mostly form *in vitro* (36). Despite less accurate binding sites from PAR-CLIP (56), all of the observations from density comparisons and conservation analyses (Figure 1) also hold for PAR-CLIP data (Supplementary Figure S5).

CLASH data are limited to abundant miRNAs and transcripts expressed in the used cell line with chimeric read throughput dictated by ligation efficiency, thus presenting only a subset of all miRNA:target interactions in human transcriptome. Our findings are based on the CLASH data; however, they may be generalizable especially if the CLASH-identified miRNA:target interactions represent a statistical sample of all interactions in human transcriptome. Genetic variants can create new predicted miRNA seed sites (19). However, it is unknown to what extent these sites are effective for miRNA binding. An analysis revealed that over 90% of seed sites were not bound according to CLIP data (52).

## SUPPLEMENTARY DATA

Supplementary Data are available at NAR Online.

SUPPLEMENTARY DATA
