# Supplementary material for: Effects of genetic variations on microRNA: target interactions
Source: Nucleic Acids Res. 2014 Jul 31;42(15):9543–52. doi: 10.1093/nar/gku675 (PMC4150780; doi:10.1093/nar/gku675)
Supplement: SUPPLEMENTARY DATA [file supp_42_15_9543__index.html]

Effects of genetic variations on microRNA: target interactions — Effects of genetic variations on microRNA: target interactions — SUPPLEMENTARY DATA 

# Chimeric bifunctional oligonucleotides as a novel tool to invade telomerase assembly

## SUPPLEMENTARY DATA

**Files in this Data Supplement:**

- SUPPLEMENTARY DATA
