# Supplementary material for: Effects of genetic variations on microRNA: target interactions
Source: Nucleic Acids Res. 2014 Jul 31;42(15):9543–52. doi: 10.1093/nar/gku675 (PMC4150780; doi:10.1093/nar/gku675)
Supplement: SUPPLEMENTARY DATA [file supp_gku675_nar-01456-z-2014-File003.zip › NAR-01456-2014 Suppl files/Supplementary_figs.pdf]

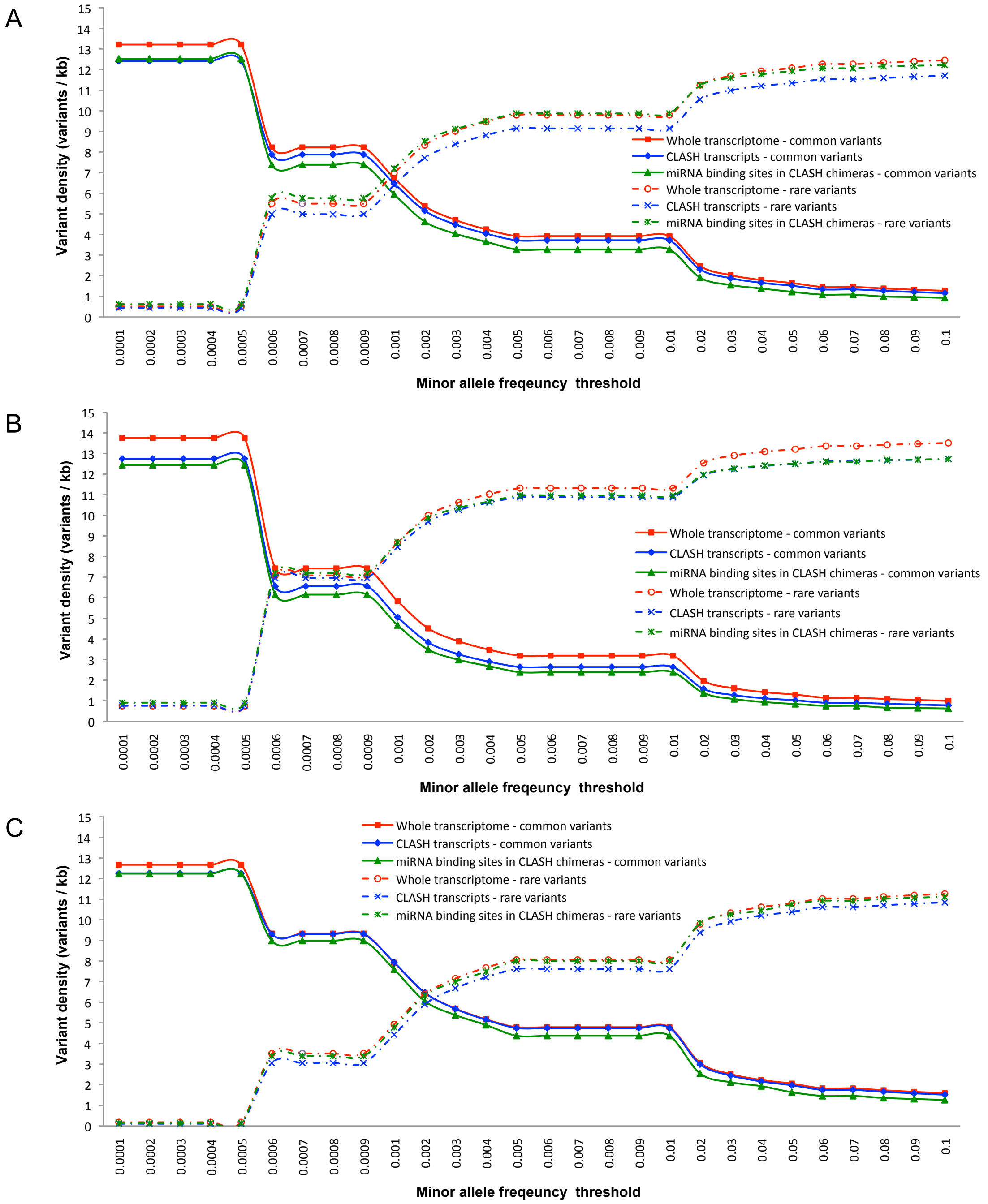

Supplementary Figure 1. Densities in whole transcriptome, CLASH transcripts and miRNA binding sites for common variants (MAF>=Θ) and rare variants (MAF<Θ) in different regions: A) mRNA; B) CDS; C) 3′ UTR, where Θ is MAF threshold and varies along the X-axis.

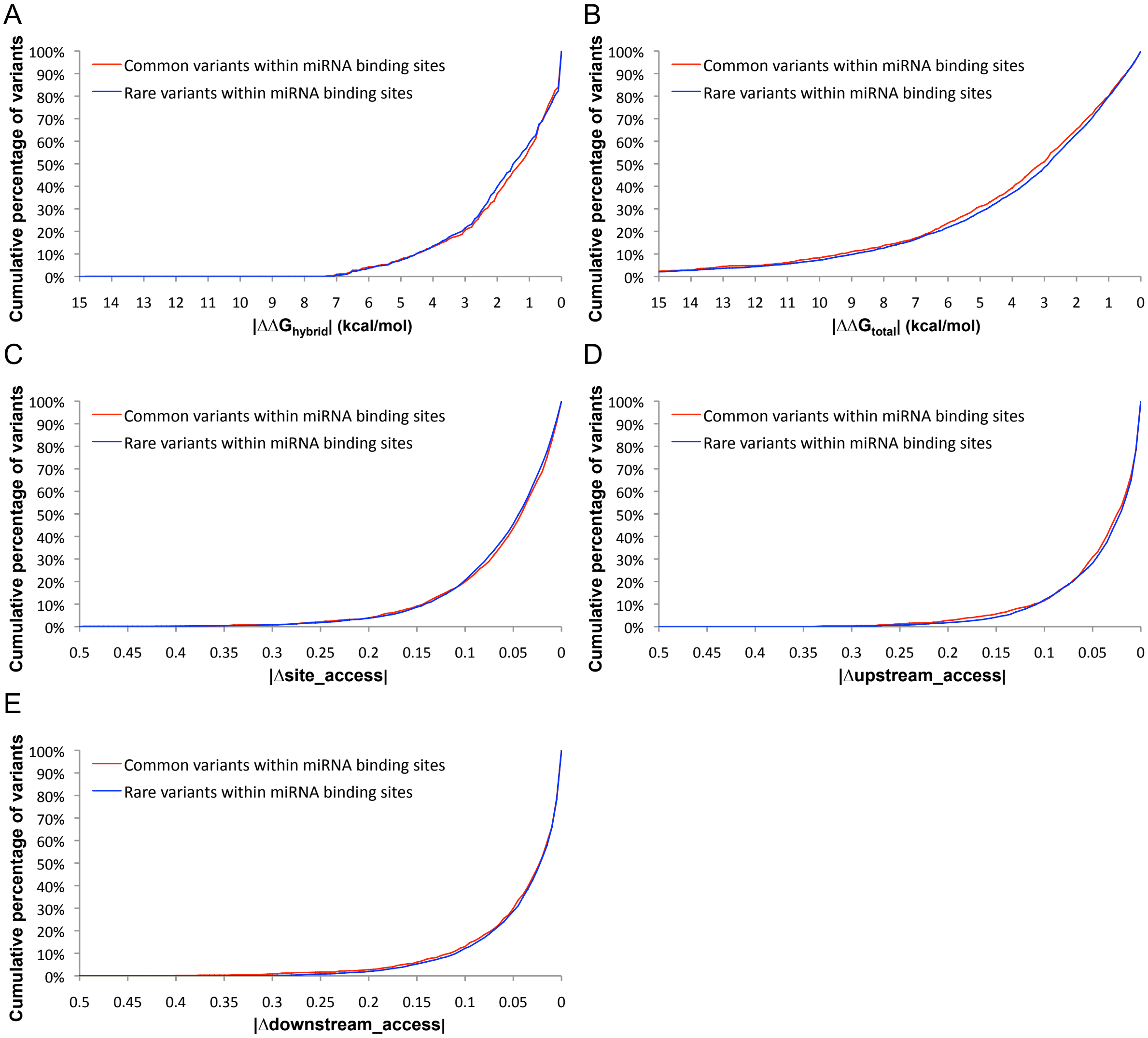

Supplementary Figure 2. The cumulative percentages of common (MAF≥1%) and rare (MAF<1%) variants in miRNA binding sites, whose absolute values of effect measure changes are greater than or equal to Θ with varying values in X-axis for **A**) $|\Delta\Delta G_{hybrid}|$; **B**) $|\Delta\Delta G_{total}|$; **C**) |Δsite_access|; **D**) |Δupstream_access|; **E**) |Δdownstream_access|.

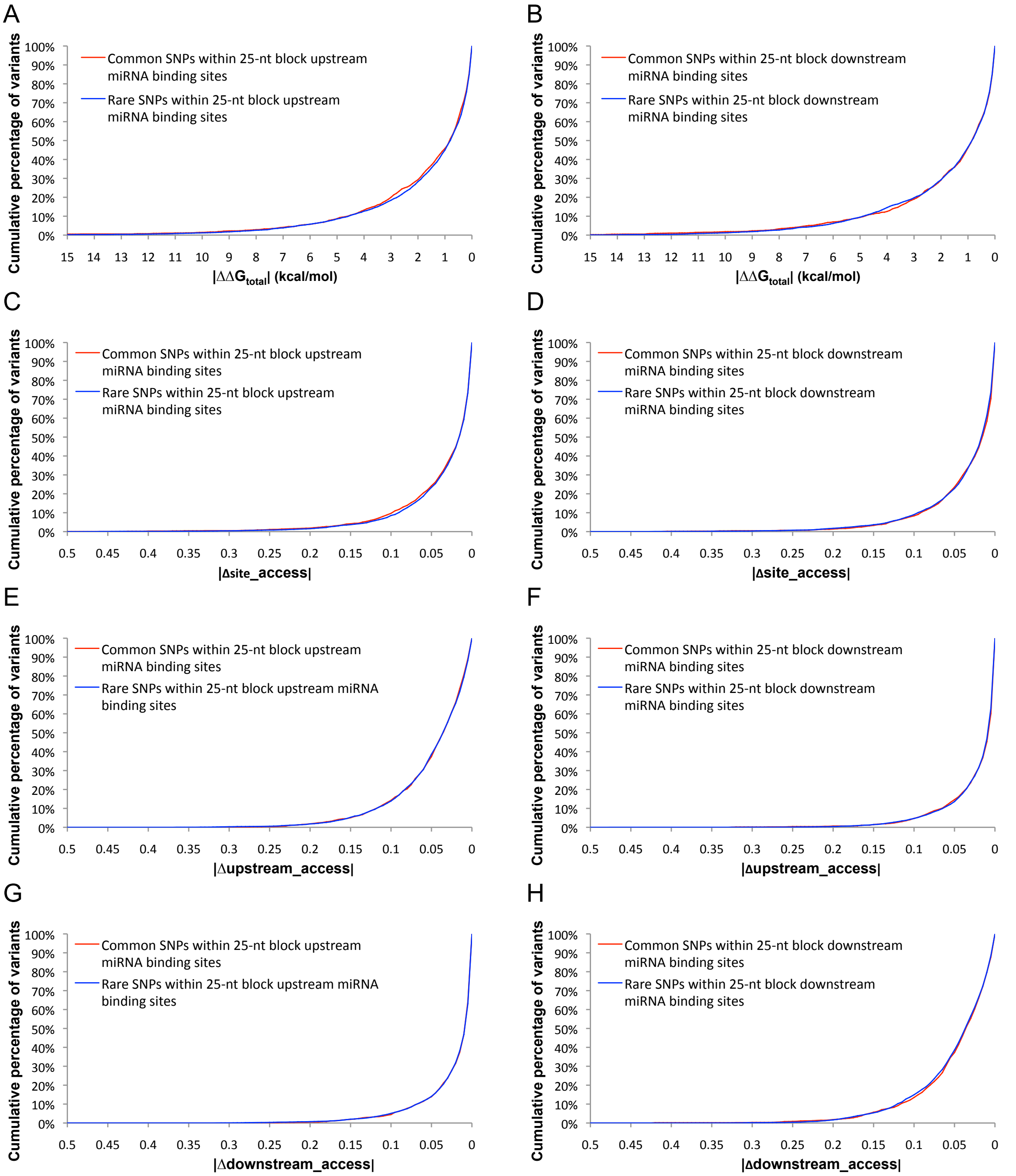

Supplementary Figure 3. The cumulative percentages of common (MAF≥1%) and rare (MAF<1%) SNPs in 25-nt blocks upstream or downstream of miRNA binding sites, whose absolute values of effect measure changes are greater than or equal to Θ with varying values in X-axis for **A**) $|\Delta\Delta G_{total}|$ for SNPs upstream of sites; **B**) $|\Delta\Delta G_{total}|$ for SNPs downstream of sites; **C**) |Δsite_access| for SNPs upstream of sites; **D**) |Δsite_access| for SNPs downstream of sites; **E**) |Δupstream_access| for SNPs upstream of sites; **F**) |Δupstream_access| for SNPs downstream of sites; **G**) |Δdownstream_access| for SNPs upstream of sites; **H**) |Δdownstream_access| for SNPs downstream of sites.

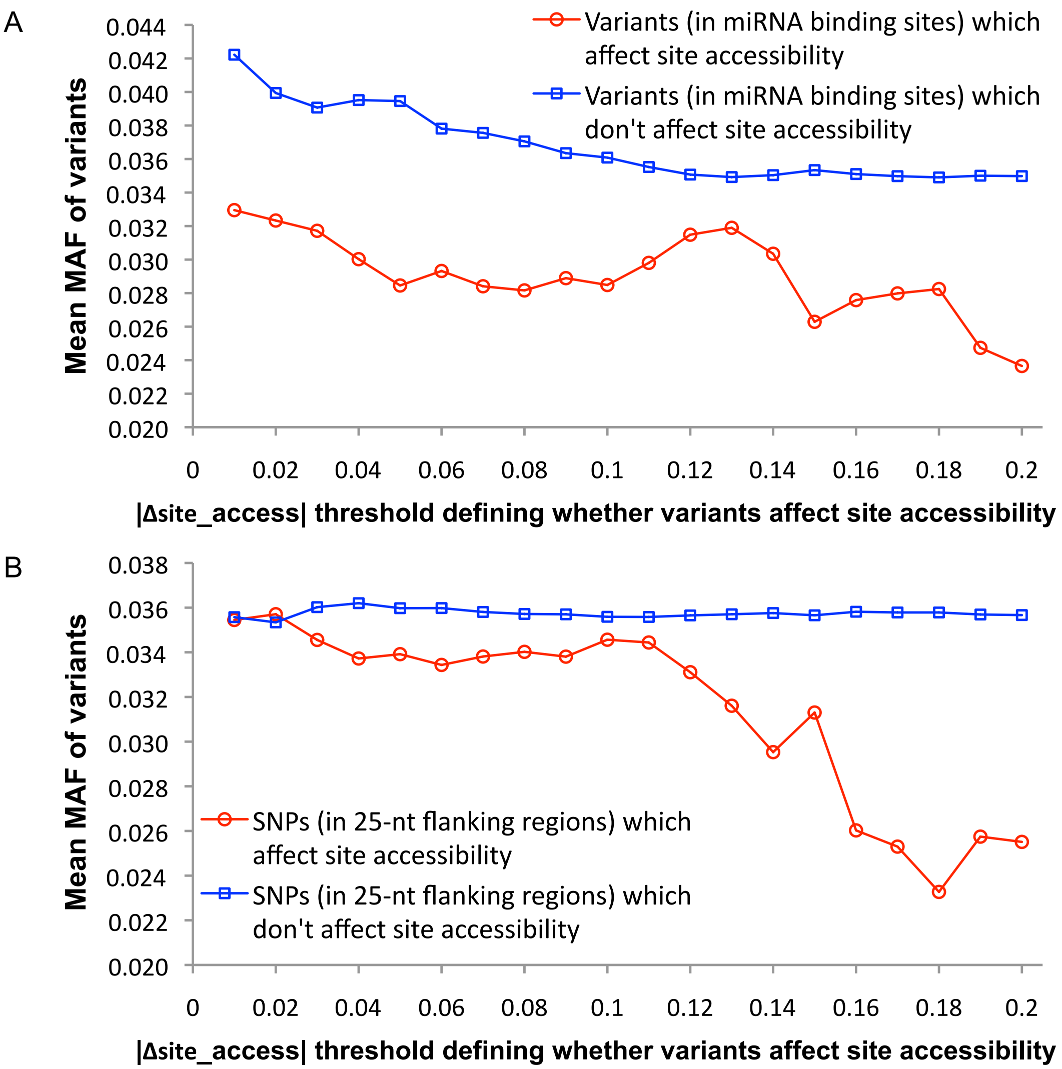

Supplementary Figure 4. Mean MAF of variants which affect site accessibility (|Δsite_access|>Θ) and those which do not (|Δsite_access|≤Θ), where Θ is |Δsite_access| threshold and varies along the X-axis. **A**) Variants in miRNA binding sites; **B**) SNPs in 25-nt flanking regions of miRNA binding sites

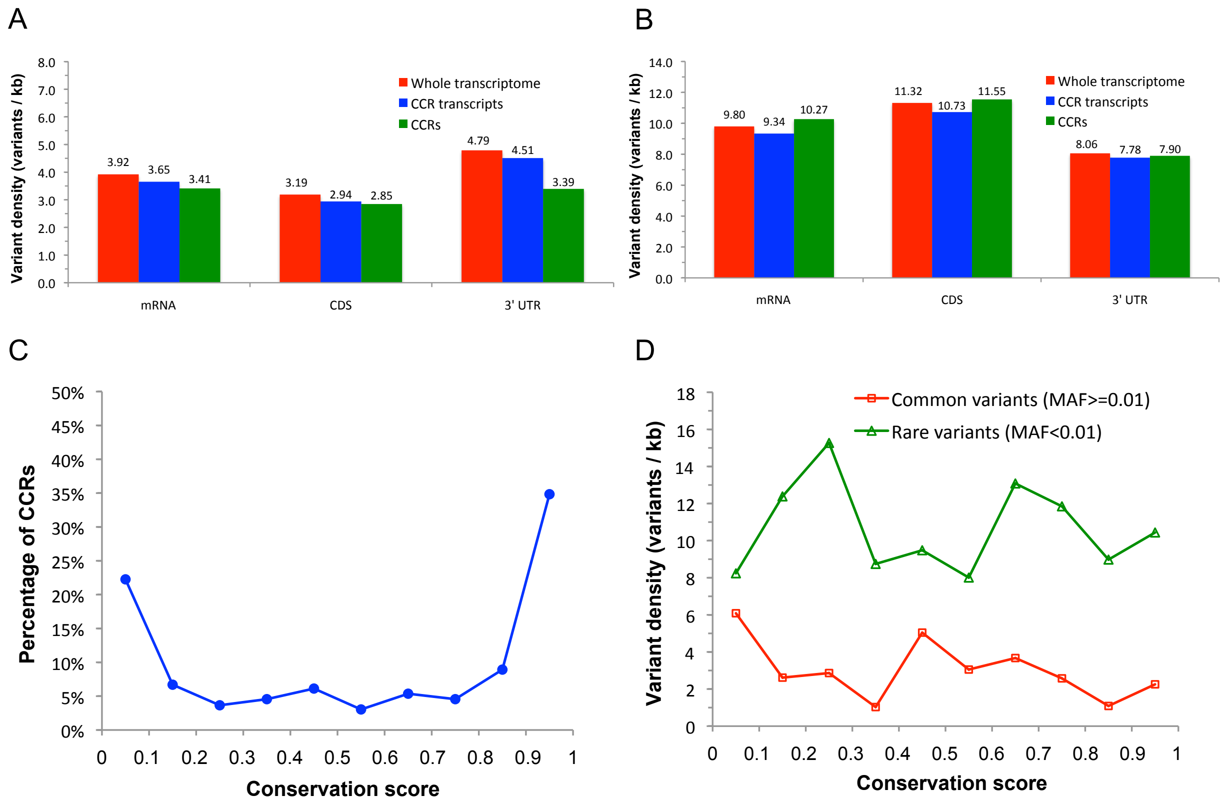

Supplementary Figure 5. Variant densities in whole transcriptome, PAR-CLIP crosslink-centered region (CCR) transcripts and PAR-CLIP CCRs for **A**) common variants (MAF≥1%); **B**) rare variants (MAF<1%); **C**) Percentages of CCRs by evolutionary conservation levels; **D**) Density of variants (common or rare) with different MAF thresholds for CCRs grouped by conservation level. The PAR-CLIP CCRs were also mapped to Ensembl transcriptome of all protein-coding genes using the annotation file (Ensembl Release 60) from Ensembl genome browser. The density computation and comparison were performed based on transcript coordinates.
